# Efficacy of different fibres and flour mixes in South-Asian flatbreads for reducing post-prandial glucose responses in healthy adults

**DOI:** 10.1007/s00394-016-1242-9

**Published:** 2016-06-21

**Authors:** Hanny M. Boers, Katrina MacAulay, Peter Murray, Jack Seijen ten Hoorn, Anne-Roos Hoogenraad, Harry P. F. Peters, Maria A. M. Vente-Spreeuwenberg, David J. Mela

**Affiliations:** 1 Unilever R&D Vlaardingen, PO Box 114, 3130 AC Vlaardingen, The Netherlands; 2 Unilever Discover R&D Colworth, Sharnbrook, UK

**Keywords:** Atta, Viscous fibre, Glycaemic response, In vitro digestion, Appetite

## Abstract

**Purpose:**

Type 2 diabetes (T2DM) is increasing, particularly in South-East Asia. Intake of high-glycaemic foods has been positively associated with T2DM, and feasible routes to reduce the glycaemic response to carbohydrate-rich staple foods are needed. The research question was whether different fibre and legume flour mixes in flatbreads lower postprandial glucose (PPG) responses.

**Methods:**

Using a balanced incomplete block design, we tested the inclusion of guar gum (GG), konjac mannan (KM) and chickpea flour (CPF) in 10 combinations (2/4/6 g GG; 2/4 g KM; 15 g CPF, and 10 or 15 g CPF plus 2 or 4 g GG) in 100 g total of a control commercial high-fibre flatbread flour mix (“atta”) on PPG in 38 normal-weight adults. Self-reported appetite was an additional exploratory outcome. An in vitro digestion assay was adapted for flatbreads and assessed for prediction of in vivo PPG.

**Results:**

Flatbreads with 6 g GG, 4 g KM, and 15 g CPF plus 2 or 4 g GG reduced PPG ≥30 % (*p* < 0.01), while no other combinations differed significantly from the control. A statistical model with four in vitro parameters (rate of digestion, %RDS, AUC, carbohydrate level) was highly predictive of PPG results (adjusted *R* ^2^ = 0.89). Test products were similar to the control for appetite-related measures.

**Conclusions:**

The results confirm the efficacy of specific additions to flatbread flour mixes for reducing PPG and the value of the in vitro model as a predictive tool with these ingredients and product format.

This trial is registered at ClinicalTrials.gov with identifier NCT02671214.

**Electronic supplementary material:**

The online version of this article (doi:10.1007/s00394-016-1242-9) contains supplementary material, which is available to authorized users.

## Introduction

The global incidence of type 2 diabetes mellitus (T2DM) is increasing at an alarming rate. Developing countries such as India have high and rapidly increasing prevalence of both pre-diabetes and diabetes [1], and consequently, there is considerable public health and consumer interest in taking steps to reduce the risk of these conditions developing and progressing. In the early state of T2DM (pre-diabetes), the condition is reversible [2]. Evidence suggests that one of the earliest events in the aetiology of T2DM is dysregulated postprandial blood glucose (PPG) [3]. Targeted reductions in PPG in (pre-)diabetic populations, using the α-glucosidase and α-amylase inhibitor acarbose, have been shown to result in significant reduction in PPG and improved long-term glycaemic control [4, 5], as well as significantly reducing the progression of pre-diabetes to T2DM and cardiovascular events [6]. The benefit of PPG lowering in the prevention of (pre-)diabetes [7] is endorsed by key professional bodies [8, 9].

Carbohydrate-rich staple foods are interesting candidates for reducing PPG exposure, because their frequent and consistent use makes them an important contributor to daily glycaemic load [10]. The two most common staples in Southeast Asia are wheat-based flatbreads and rice [11]. Flatbreads are typically prepared at home from a commercially-made whole-wheat flour mix (“atta”). Commercially-viable, efficacious routes to reduce the PPG response to flatbreads are therefore of interest.

Soluble viscous fibres are known to lower PPG, mainly by reducing the rates of gastric emptying and glucose absorption in the intestine [12]. In addition, legume flours, such as chickpea flour (CPF), are known to give a flatter blood glucose response than wheat flours [13]. Previous research has shown that soluble viscous fibres (viz., beta-glucan, psyllium and fenugreek*)* with or without legume flour can lower the PPG of flatbreads [14–16]. While there are no human studies on the combination of guar gum (GG) and CPF, a study in rats showed that a combination of 5 % CPF and 1 % GG gave a reduction in fasting blood glucose superior to 2 or 3 % GG alone [17]. As the addition of high levels of viscous fibres may have adverse effects on flatbread mix cost and sensory attributes, routes to achieve efficacy at lower fibre additions are of interest. However, to date there is no clinical study which systematically tested the potential for different soluble fibres with or without legume flour to lower the PPG response to flatbreads. A further question is whether GG could partly be replaced by CPF in flatbreads to achieve reductions in PPG similar to a higher GG level alone. This research was therefore undertaken as a first step in a programme to find efficacious, but also affordable and acceptable routes to lower the PPG response to commercial flatbread mixes.

From the existing literature, additions of konjac mannan (KM) and of GG alone or combined with CPF were prioritized for potential feasibility and efficacy in clinical testing [17–21]. The primary objective of this study was to identify one or more flour compositions that gave a significant difference in the positive incremental area under the 2-h curve (+iAUC_2hr_) for plasma glucose after consumption of the test relative to the control product. Exploratory objectives were to estimate the maximum observed glucose response (*C*_max_), the time at which the *C*_max_ was reached (*T*_max_) and the mean plasma glucose level at 3 h. As soluble viscous fibres and CPFs are also claimed to increase satiety [22, 23], an additional exploratory objective was to assess possible effects on appetite-related parameters. Finally, an in vitro digestibility assay specifically adapted for flatbreads was developed to assess how well this predicted the observed in vivo results in this product format.

## Methods

### Test product and preparation

The research was based on 12 test products containing 100 g flour per serving. An existing commercial fibre-enriched (high-fibre flour, HFF) commercial mix (market standard atta, Hindustan Unilever Ltd., India) containing whole wheat flour with 5 g bran per 100 g was used as the control. The 11 products tested against this included a “market standard” product (no added fibres) and 10 experimental products based on the HFF control with the inclusion of 10 or 15 g CPF, 2 or 4 g KM, 2, 4 or 6 g GG per 100 g flour in combinations shown in Table 1. The viscosity of the GG and KM was tested and verified, and these data are also reported in Table 1. All flour mixes were formulated by the research sponsor (Unilever R&D, Vlaardingen, The Netherlands). For clinical testing, flatbreads were prepared fresh at the test site. For each single test serving, 100 g flour was kneaded to a soft and uniform consistency with the addition of ~73 ml water and allowed to rest for 30 min and then divided into 3 equal balls and rolled to 2–3 mm thickness. More water was added as needed to achieve the desired texture when fibres or legume flour were incorporated (see Table 1). Flatbreads were subsequently baked and kept warm until consumption within 30 min of cooking or used for in vitro analysis.

**Table 1:** Composition of test flatbreads: available carbohydrates, dietary fibre and water

Flatbreads	Composition^a^	Total available carbs (g)	Total dietary fibre (g) (AOAC 2009.01)	Water (% weight)
HFF (control)	100 g high-fibre flour (HFF)	61	11	34.5
1	81 g HFF + 15 g CPF^b^ + 4 g GG^c^	55	16	37.1
2	83 g HFF + 15 g CPF + 2 g GG	56	14	34.6
3	85 g HFF + 15 g CPF	57	13	30.6
4	86 g HFF + 10 g CPF + 4 g GG	56	15	36.8
5	88 g HFF + 10 g CPF + 2 g GG	57	14	34.5
6	94 g HFF + 6 g GG	57	16	39.7
7	96 g HFF + 4 g GG	59	14	37.2
8	98 g HFF + 2 g GG	60	13	36.8
9	96 g HFF + 4 g KM^d^	59	14	39.2
10	98 g HFF + 2 g KM	60	13	37.9
11	Market standard atta	64	8	33.6

^a^ *HFF* high-fibre flour (control), *CPF* chickpea flour, *GG* guar gum, *KM* konjac mannan

^b^Chickpea flour (Avent Agro Pvt. Ltd., Delhi, India)

^c^Guar gum (Ace Gum Industries PVT. LTD, Mumbai, India); viscosity cold 1 % in water, measured by a Brookfield RVF viscometer 20-RPM Spindle no. 4, at 30 min: 4500 mpa.s, at 2 h 5400 CPS and 24 h: 5500 mpa.s

^d^Konjac mannan (Hubei Konson Konjac Gum Co., LTD., Wuhan Hubei, China); viscosity 25 °C, 1 %, mix round half an hour, measured by model NDJ-1 viscometer, spindle 4#, 12-RPM. Test after an hour dissolved; >22,000 mpa.s)

### Human study

#### Participants

Seventy-three apparently healthy volunteers were recruited for screening from an existing database of potential participants in the local area of Leatherhead Food International (Leatherhead, UK), where the study was conducted. For the detailed selection criteria, see Supplemental Table 1 (Online Resource). The study was conducted according to the principles of Good Clinical Practice, the Declaration of Helsinki (2008) and applicable local laws and regulations concerning studies conducted on human subjects, not testing a medical product or device. Ethical approval for the study was obtained from the East Kent Local Research Committee. Each participant provided written informed consent prior to his/her inclusion in the study.

##### Experimental design

This study used a double-blind, randomized, balanced-order incomplete block design. Randomization to treatment orders was executed using a computer with a random number generator by a second statistician, who was not involved in the study. All persons involved in the study were blinded. All subjects received the control (HFF) and 4 out of the 11 other test products. For this study design, a power calculation indicated that a minimum of 14 subjects per test product would be required to test for the significance of a 30 % reduction in +iAUC_2hr_ (the area of the PPG response lying above the baseline concentration) vs the control product, assuming a standard deviation of 32.7 mmol/l.min (based on previous studies at the test site), at *α* = 0.20 and *β* = 0.80. With this design and 42 subjects in the study, all of the test products would be tested on at least 15 participants (8 products tested by 15 subjects, 3 products tested by 16 subjects and all subjects getting the control).

Subjects attended the initial screening day followed by 5 test days, at least 1 week apart. They were instructed to minimize changes in their diet and activity during the test period. On the day prior to each test day, each subject was instructed to refrain from physical activity and alcohol consumption and to consume their same evening meal. All participants fasted overnight (from 20.00 h until consumption of the test product), but were allowed to drink water ad libitum. At time = 0 min on each test day, subjects consumed three freshly made flatbreads (100 g flour total) with 250 ml water as breakfast and completed this within a 15-min period at every visit at the same time and day of the week. They were allowed to drink up to 150 ml water every subsequent hour, to be consumed after finger pricks and self-reported appetite ratings. The volume of water consumed was registered.

##### Blood collection and glucose measurements

Capillary blood was collected by finger prick into lithium heparin and sodium fluoride tubes for plasma glucose analysis. Three basal samples were collected at −15 min and then at 15, 30, 45, 60, 90, 120 and 180 min after the test meals. All samples were centrifuged (3000 rpm for 10 min at 4 °C) prior to immediate analysis or storage at −20 °C. Plasma glucose concentrations were measured on a YSI 2300 STAT Plus™ Glucose and Lactate Analyzer (YSI Life Sciences).

##### Measurement of appetite

Self-ratings of appetite feelings (“how hungry are you”, “how full are you” and “how strong is your desire to eat a meal”) were made at baseline (pre-consumption) and 15, 30, 60 and 120 min post-prandially. These were scored by means of a mark on a 60-mm Electronic Visual Analogue Scale (EVAS) [24] on a pocket PC (iPAQ), anchored at the low and high end with “not at all” and “extremely” [25].

#### In vitro determination of starch digestibility

Starch digestibility in vitro was assessed by an adaptation of the Englyst method [26, 27] which has been demonstrated to show good correlation between PPG responses and in vitro starch digestibility in terms of rapidly digestible starch (RDS), slowly digestible starch (SDS) and resistant starch (RS) for a wide range of products [28]. The method was modified with the methods described by Sopade [29] and Van Kempen [30] to provide a glucose release profile describing how much glucose is released per unit of time. Briefly, sliced pieces of chapatti (500 mg) were mixed for 30 s with an α-amylase solution (1 ml, 300 U Sigma 10080) to simulate starch digestion in the mouth. Gastric digestion was simulated by 30-min incubation at 37 °C in pepsin solution (5 ml, 16 kU Sigma P77160 in 0.05 M HCl). After neutralization with NaOH, intestinal digestion was simulated by 4-h incubation at 37 °C in pancreatic solution (30 ml, 56 mg Sigma P1625 + 100 U Sigma A7095 + 1 kU invertase in 0.2 M acetate buffer pH 6.0). Aliquots (200 µL) from this phase were collected into ethanol at 0, 20, 60, 120, 180 and 240 min, to stop further enzyme activity, and analysed for glucose (Sigma Glucose assay kit GAGO20). The average (*n* = 2) glucose data were fitted with the Chapman–Richards model to give the rate of digestion (*k*) and the AUC_120_ using the trapezoidal model (See Online Resource for further explanation).

##### Statistical methods

The primary outcome variable was plasma glucose +iAUC_2hr_. This was calculated using the trapezoidal rule, and linear interpolation was used to establish the time of crossing between time points where the PPG crossed the baseline value. Statistical comparisons were only made between the control and other test products using a mixed model analysis of variance, with subject as a random effect and product as a fixed effect. Baseline (fasting score) for each product was included as a covariate, as was the average baseline per subject over all products, the latter to avoid bias in the product estimates due to the mixed model. Gender, body weight and the order of product testing were all included as covariates. Dunnett’s test was used to adjust for the multiple comparisons using an overall significance level of 0.20. All analyses were performed with SAS version 9.2 (SAS Institute, Cary, NC, USA). Exploratory variables included the maximum postmeal plasma glucose concentration (*C*_max_), time when this was reached (*T*_max_), mean plasma glucose at 3 h and self-reported scores on the 3 appetite-related measures. The AUC for appetite ratings scales was calculated using the trapezoidal rule and expressed as the original scale units by dividing by the length of time measured. There were no pre-planned statistical analyses of exploratory measures, and therefore, only descriptive statistics are presented for these. The relationship between in vitro and in vivo measures was analysed with a regression model. Initial correlations and scatter plots for each of these variables with +iAUC showed that a linear model was unlikely to provide good prediction, so a quadratic model was used.

## Results

### Subject baseline characteristics

**Fig. 1:**
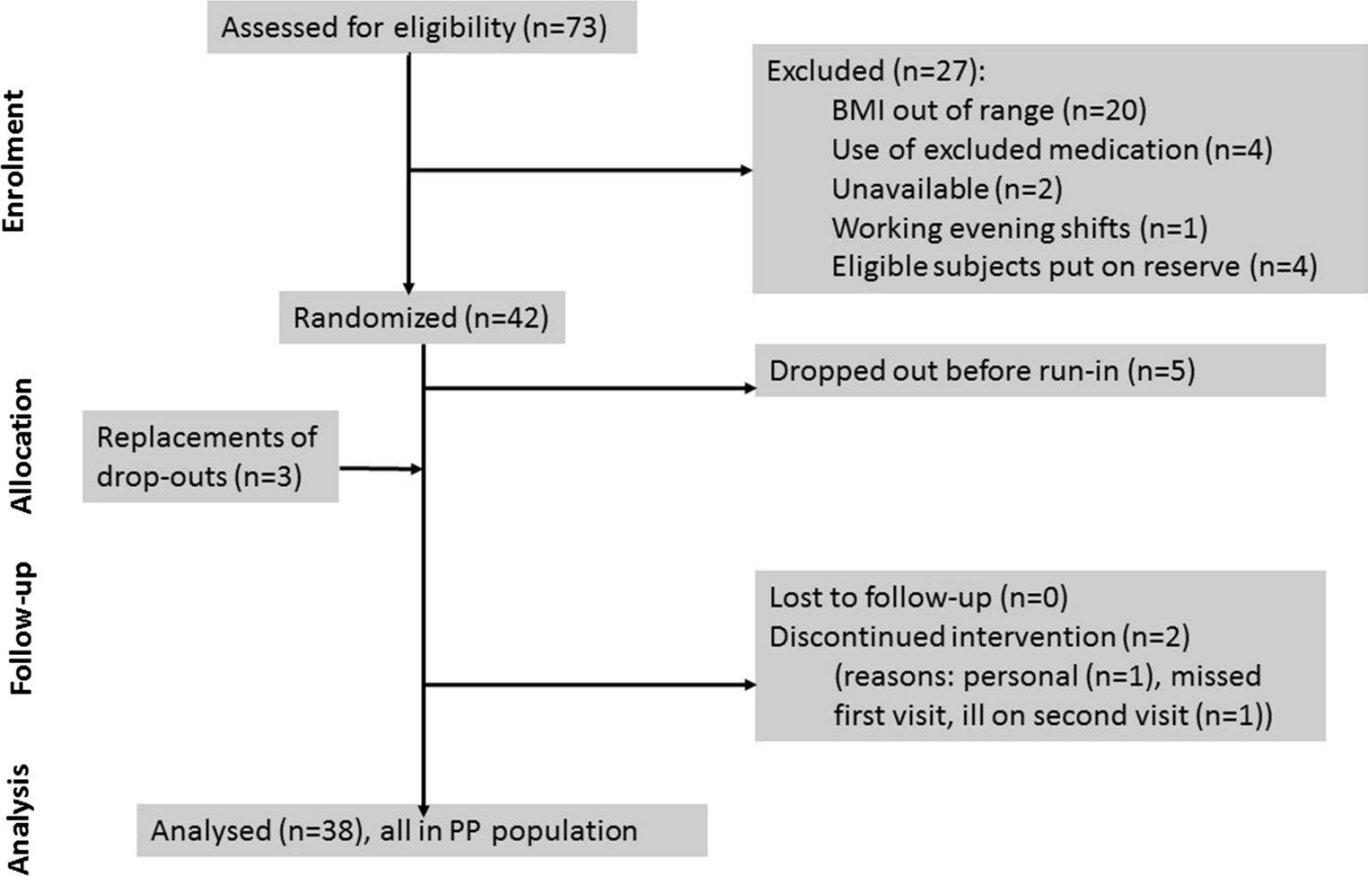
Flow diagram of participants throughout the study

From the initial 73 subjects screened for the study, 27 were excluded and 38 subjects completed the study (see Fig. 1), leaving it slightly below the planned power for most test products (9–14 subjects per product). The baseline characteristics of participants are shown in Table 2 (and separately by gender in the Online Resource information supplemental Table 2).

**Table 2:** Subject baseline demographic characteristics, mean ± standard deviation

	Subject baseline demographic characteristics (mean ± SD)
Age (year)	37 ± 9
Gender (male/female)	3/35
Height (m)	1.67 ± 0.06
Body weight (kg)	64.1 ± 8.7
BMI (kg/m^2^)	22.8 ± 1.6
Fasting plasma glucose (mmol/l)	5.1 ± 0.4

### Postprandial plasma glucose concentrations

**Fig. 2:**
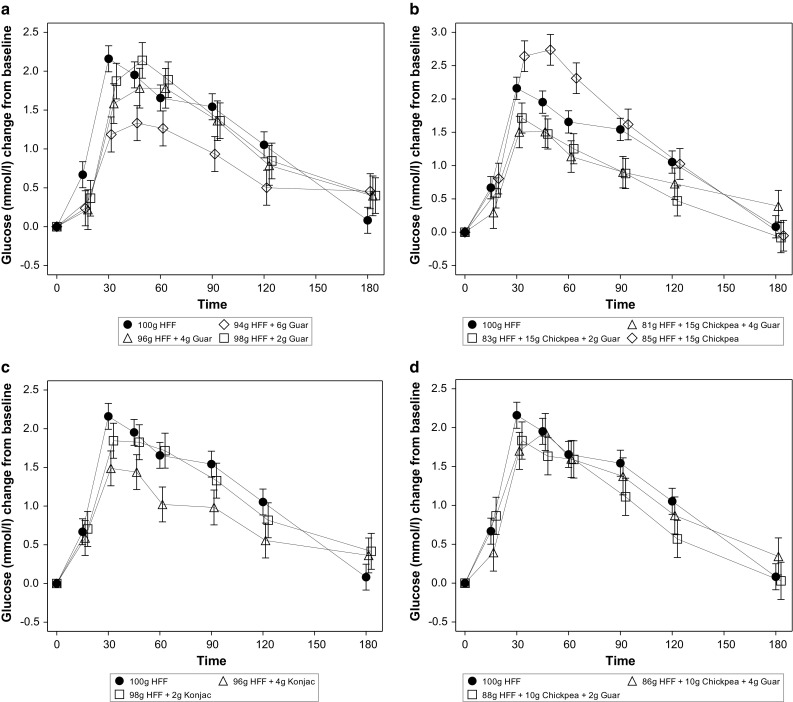
Effect of flatbreads consumption with different amounts of viscous fibres and legume flour on postprandial glucose (mean ± SEM) (*HFF* high-fibre flatbread control). **a** Effect of flatbread consumption with different amounts of guar gum on postprandial plasma glucose (mean ± SEM) (*HFF* high-fibre flatbread control). **b** Effect of flatbread consumption with different amounts of chickpea flour (15 g) without or with guar gum on postprandial plasma glucose (mean ± SEM) (*HFF* high-fibre flatbread control). **c** Effect of flatbread consumption with different amounts of konjac mannan on postprandial plasma glucose (mean ± SEM) (*HFF* high-fibre flatbread control). **d** Effect of flatbread consumption with chickpea flour (10 g) and different amounts of guar gum on postprandial plasma glucose (mean ± SEM) (*HFF* high-fibre flatbread control)

**Fig. 3:**
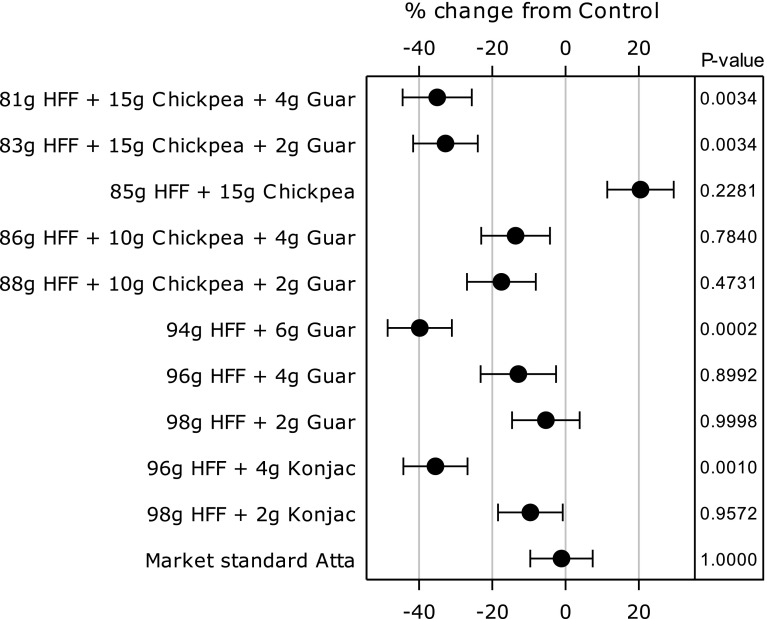
Percentage change (mean ± SEM) in PPG (+iAUC_2hr_) of flatbreads with different amounts of viscous fibres and/or legume flour and *p* value for change relative to the control flatbread without additions of viscous fibres or legume flour

Fasting plasma glucose values were similar within test product groups and also within subjects on different test days (data not shown). The observed PPG response patterns for addition of GG, KM and CPF or combinations of CPF + GG are shown in Fig. 2a–d. Data for per cent differences in plasma glucose +iAUC_2hr_ vs control are shown in Fig. 3, and the absolute values are given in Table 3. Postprandial plasma glucose +iAUC_2hr_ was statistically significantly reduced from the control HFF in 4 test products: 15 g CPF + 4 g GG, 15 g CPF + 2 g GG, 6 g GG and 4 g KM (Table 3; Fig. 2). The data show a general dose–response reduction in +iAUC_2hr_ with 2 and 4 g KM and 2, 4 and 6 g GG relative to control, with the addition of 10 g CPF to 2 and 4 g GG having little further effect. While addition of 15 g CPF alone led to a non-significant increase in PPG and the use of 2 g GG and 4 g GG non-significantly decreased PPG, the combination of 15 g CPF and 2 or 4 g GG led to marked and significant reductions in glucose +iAUC_2h,_ exceeding a 30 % reduction (Fig. 3).

**Table 3:** Glucose response curve analysis (mean absolute change from control ± SEM)

	Flatbread composition	Glucose +iAUC_2hr_ (absolute and [%] difference from control (169.34 mmol/l.min))	Glucose *C*_max_ (absolute and [%] difference from control (7.65 mmol/l))	Glucose *T*_max_ (difference from control (40.59 min))	Glucose at t = 180 min (difference from control (5.30 mmol/l))	Number of participants for study arm
1	81 g HFF + 15 g CPF + 4 g GG	−59.34 ± 15.99 * [−35.04 ± 9.44]	−0.64 ± 0.21 [−8.37 ± 2.68]	5.62 ± 5.95	0.32 ± 0.16	11
2	83 g HFF + 15 g CPF + 2 g GG	−55.51 ± 14.94* [−32.78 ± 8.83]	−0.52 ± 0.19 [−6.85 ± 2.51]	−2.71 ± 5.55	−0.14 ± 0.15	13
3	85 g HFF + 15 g CPF	34.64 ± 15.36 [20.46 ± 9.07]	0.55 ± 0.20 [7.16 ± 2.58]	3.62 ± 5.71	−0.07 ± 0.15	12
4	86 g HFF + 10 g CPF + 4 g GG	−23.11 ± 15.90 [−13.65 ± 9.39]	−0.31 ± 0.20 [−4.02 ± 2.67]	6.96 ± 5.91	0.21 ± 0.16	11
5	88 g HFF + 10 g CPF + 2 g GG	−29.67 ± 15.91 [−17.52 ± 9.39]	−0.37 ± 0.20 [−4.80 ± 2.67]	3.84 ± 5.90	−0.01 ± 0.16	11
6	94 g HFF + 6 g GG	−67.41 ± 14.86* [−39.81 ± 8.77]	−0.97 ± 0.19 [−12.69 ± 2.49]	5.96 ± 5.53	0.40 ± 0.15	13
7	96 g HFF + 4 g GG	−21.84 ± 17.45 [−12.90 ± 10.30]	−0.60 ± 0.22 [−7.80 ± 2.93]	7.04 ± 6.50	0.34 ± 0.17	9
8	98 g HFF + 2 g GG	−9.10 ± 15.63 [−5.38 ± 9.23]	−0.09 ± 0.20 [−1.15 ± 2.62]	5.97 ± 5.81	0.38 ± 0.15	12
9	96 g HFF + 4 g KM	−60.16 ± 14.83* [−35.53 ± 8.76]	−0.74 ± 0.19 [−9.63 ± 2.49]	−1.37 ± 5.53	0.36 ± 0.15	13
10	98 g HFF + 2 g KM	−16.28 ± 14.95 [−9.61 ± 8.83]	−0.18 ± 0.19 [−2.31 ± 2.51]	7.64 ± 5.56	0.35 ± 0.15	13
11	Market standard atta	−1.89 ± 14.43 [−1.12 ± 8.52]	−0.10 ± 0.19 [−1.29 ± 2.42]	5.56 ± 5.37	0.25 ± 0.14	14

*HFF* high-fibre Annapurna flour, *CPF* chickpea flour, *GG* guar gum and *KM* konjac mannan

* Statistically significant, *p* < 0.05 versus control

### Exploratory outcomes

*C*_max_, *T*_max_ and 3-h plasma glucose data are shown in Table 3. *C*_max_ data were largely consistent with the +iAUC data, while *T*_max_ was in general little different from the control. Mean plasma glucose at 3 h for most treatments was mildly raised relative to the control. Appetite data were similar for all test products and suggest no consistent effects relative to the control (See Supplemental Fig. 2a–c (Online Resource)).

#### Adverse events

There were 5 adverse events that possibly could be considered related to the study procedures: 2 times nausea with vomiting, 2 times nausea alone and once with flatulence. Nausea occurred only in conjunction with GG. The participants reporting adverse events were excluded from the statistical analysis.

### In vitro measures of starch digestibility

**Fig. 4:**
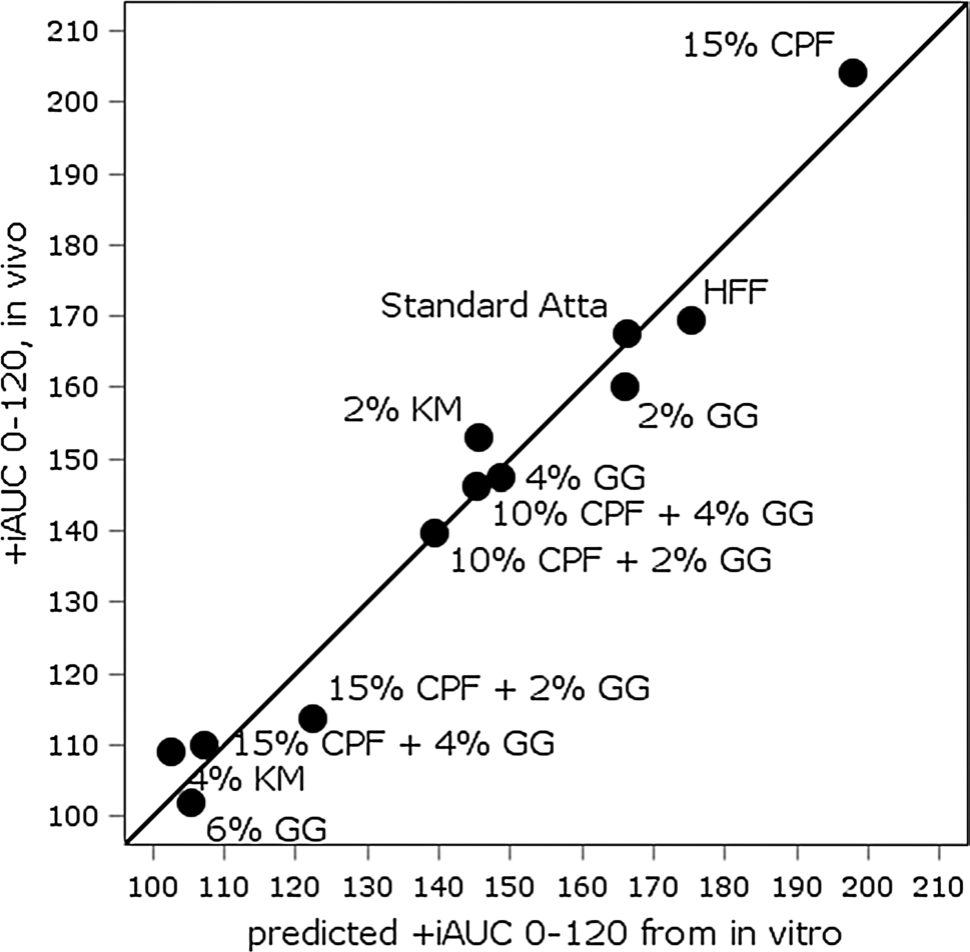
Observed in vivo response (+iAUC) for postprandial plasma glucose response versus the value predicted based on in vitro data (statistical model as described in text and Online Resource). *HFF* high-fibre flour (control), *CPF* chickpea flour, *GG* guar gum and *KM* konjac mannan

The separate measures alone from the in vitro assay, such as RDS, SDS, RS, *k* and AUC, individually had inconsistent relationships with the dose and types of added fibres and likely to be poor predictors for the in vivo +iAUC. A model based on just the Englyst [26, 27] parameters (RDS and RS) also had a poor predictive value (see Online Resource). In contrast, a statistical model comprised of the in vitro parameters *k* (for rate of digestion), %RDS, AUC in vitro (=AUC for 120 min) and CHO (=carbohydrate level) was highly predictive of the observed clinical PPG results (*R* ^2^ = 0.97 and adjusted *R* ^2^ = 0.89) (see Fig. 4). The resulting model was:

$$\begin{aligned} + {\text{iAUC}} &= 31,417 + 334.6*{\text{CHO}} - 9729 \\ & \quad *k - 4.739*{\text{AUC}}\_{\text{in vitro}} - 32.03*{\text{RDS}} \hfill \\ \quad + 18.38*{\text{CHO}}*k*1.183*k*{\text{AUC}}\_{\text{in}}\,{\text{vitro}}\\ & \quad {-}3.542*{\text{CHO}}^{2} {-}132.2*k2 \hfill \\ \end{aligned}$$

where *k* = rate of digestion from Chapman–Richards model; AUC in vitro = AUC for 120 min; CHO = carbohydrates content of flour mix; RDS = rapidly digestible starch [see Supplemental Table 5 (Online Resource)].

## Discussion

This research demonstrates that several flatbread flour mix (atta) formulations incorporating GG, KM and CPF significantly lower PPG levels relative to a commercial product with added bran fibre. These findings build on previous reports demonstrating that incorporation of viscous dietary fibres and/or legume flours into other staple flatbread foods from India (chapatti, naan, rotis) can lower the PPG response after a meal [14–16]. A novel result is the demonstrated efficacy of KM in this format and the combination of CPF with low levels of GG in human subjects, as efficacy of this combination previously had been shown only in animals [17]. This suggests the potential for affordable, efficacious formulations with lower levels of GG, which may mitigate its adverse sensory impact.

We chose to measure the area under the curve and particularly the +iAUC, because this has frequently been used in human intervention studies (e.g. testing variation in glycaemic index (GI)) [31]. In addition, the +iAUC describes the glycaemic response to foods more accurately [32] than the total AUC. The *C*_max_ data are also largely consistent with the +iAUC, which is to be expected, and a reduced *C*_max_ is also seen as clinically beneficial [33]. The *C*_max_ data further suggest that the lowering of the iAUC is not so much due to a higher insulin response [34], but more likely due to other processes, e.g. rate of uptake. In contrast, *T*_max_ data are similar amongst treatments. The plasma glucose level at three hours (see Table 3) was mildly increased for the flatbreads containing viscous fibres compared to the control, which may reflect a slower digestion of starch and thus continued absorption of glucose from the intestine, as well as a lower insulin response and thus slower return of glucose to fasting levels. Although that effect is small, it would contribute to reduced glycaemic variability, which is seen as a risk factor for diabetes [3].

Although a general dose–response effect for the addition of 2, 4 and 6 g of GG alone was observed in this study, the effect of 6 g GG was much larger and statistically significant compared to the control product. Other studies have shown the efficacy of GG for lowering PPG [19, 20, 35, 36]; however, a general threshold and dose–response level cannot be determined from those studies, partly because different sources of GG (e.g. different molecular weights and chain lengths) were used and in different food formats. There are, however, a number of studies testing GG incorporated into bread, and these gave results broadly similar to the GG outcomes here. Wolever et al. [36] showed that 5 g GG in bread (10 % of the carbohydrate component) lowered the +iAUC by 42 %, while Gatenby et al. [35] observed that 7.6 g GG with different molecular weights reduced the +iAUC by 23–27 %. Those results were also reflected in data from Wolf et al. [20], who showed that 5 g GG in a drink (containing 25 g maltodextrin), consumed together with bread, lowered the +iAUC by 24 %. Lastly, addition of medium-weight GG (3.8 g, 9 g and 14.8 g) to breads containing high-amylose whole grain corn flour produced a dose-related reduction in glycaemic response [19].

The potential mechanisms of action of viscous soluble fibres such as GG and KM on PPG can be very diverse, ranging from reducing the rate of gastric emptying to reducing starch digestion and absorption in the intestine resulting in a lower PPG and insulin response [12]. The viscosity generated by plant gums is a function of the concentration of the dissolved gum and its molecular weight (MW) [37]. In this study, we used native GG, but commercially this can be hydrolysed into GG with lower MWs and correspondingly lower viscosity in the mouth as well as gastrointestinal tract, often resulting in a decreased effect on PPG [34, 35, 38, 39]. There are also indications that some viscous fibres (e.g. GG) can directly inhibit digestive enzymes [40]. On the product level, viscous fibres can also alter the rheological and/or microstructural properties of the food, resulting in reduced ability of the starch to gelatinize during cooking [41]. Viscous fibres are believed to compete with starch for water in food formats resulting in decreased gelatinization [42]. In addition, GG galactomannans may form a “barrier” around starch granules, making them resistant to enzymatic hydrolysis [43].

KM, another viscous fibre, was also introduced into this study to test the influence of viscosity on PPG. Chearskul et al. [44] evaluated the effect of 1 g KM in a capsule given 30 min before an oral glucose tolerance test in 20 diabetic subjects. At 60 min, the blood glucose concentration with KM was 7 % higher than in the control group, while at 120 min there was no difference between the two groups. The lack of any apparent beneficial effect in that study can possibly be explained by the matrix. The capsules, although taken with water, may not fully develop their viscosity under gastrointestinal conditions due to the time required for the fibre to fully hydrate. Doi [18] showed that 3.9 g KM mixed into soup decreased the rise in blood glucose at 30 min after consumption by about 30 % versus the control.

In our study, the addition of 4 g KM had nearly the same effect as 6 g GG, and this can likely be attributed to the higher viscosity of KM compared to GG (see footnotes to Table 1). KM is a glucomannan, while GG is a galactomannan. Compared with other native dietary fibres at similar concentrations, glucomannan has the highest viscosity and MW, ranging from 200 to 2000 kDa, depending upon the origin, method of processing and storage time [45]. Others have reported a highly significant inverse relationship between the peak blood glucose response and the log viscosity of drinks differing in the amounts of viscous fibre [46]. In the present study, only the highest level of 4 g KM had a substantial and statistically significant effect and not the 2 g addition, which was comparable in viscosity to the 4 g guar gum. A possible explanation is that the KM may not have been fully viscosified under gastrointestinal conditions, due to the lower solubility of KM in the solid food matrix.

In addition to increasing viscosity, replacement of wheat flour by legume flour could also have a beneficial effect on PPG. There is some evidence that CPF can lower the PPG due to its higher content of resistant starch [47] and high concentration of slowly digestible starch [48]. Zafar et al. [21] found that supplementation of whole wheat bread with 35 % (but not 25 %) CPF significantly reduced the glycaemic response. This result was also corroborated by Johnson et al. [49] in white bread. In contrast to the fact that 15 g CPF in combination with 2 and 4 g of GG markedly and significantly reduced PPG, we found that the inclusion of 15 g CPF alone in the flatbreads (though not significantly) increased PPG. The reason for the latter could be that fine grinding of legumes (as is the case for CPF) disrupts the cell structure and renders starch more readily accessible for digestion [50].

We found little apparent effect of the tested additions on self-reported appetite ratings. Other research suggests that changes in blood glucose *per se* may have limited effects on appetite [51, 52]. Clark and Slavin [53] furthermore described in a systematic review that most fibres do not reduce appetite in acute study designs. Nevertheless, there are a number of reports of enhanced satiety effects associated with the addition of legumes or specific fibres to foods and beverages [22, 23]. A systematic review of randomized controlled trials showed that GG (mean fibre dose: 10.7 g) led to significantly reduced appetite ratings in half of the observed comparisons in the literature [23]. Studies with preloads of KM before a meal did not produce significant effects on hunger, fullness or appetite scores [54, 55]. Addition of 25 or 35 % CPF to whole wheat bread also did not decrease appetite scores [21]. While a recent meta-analysis of acute feeding trials reported that dietary pulses (beans, peas, chickpeas and lentils) produced on average a 31 % greater satiety iAUC [22], the doses used varied substantially, ranging from 7.6 to 311 g (median, 160 g).

While this study used Caucasian subjects, the results are likely to also be applicable (and even more relevant) to other populations, such as those in Asia. Asian populations in general have a higher PPG response than Caucasians [56], and lowering GI is more relevant for people with poor metabolic control [57].

The in vitro digestibility method used here showed a very high correlation with in vivo PPG results. Several other studies have found a good correlation between the in vitro digestion of breads and the in vivo glycaemic response [16, 58–60]. Our method is based on that of Englyst [26, 27], but modified to reflect more realistic physiological conditions. The modification by Sopade [29] was introduced to simulate oral digestion by mixing the food with pancreatic alpha-amylase for 30 s. Another possible improvement in the Englyst method is the optimization of the intestinal pH and its amount for optimal functioning of the enzymes (see Online Resource). A final modification of the Englyst method was the extension of the number of data points to determine the rate of digestion of slowly digestible starches. By doing this, the in vitro starch digestibility curve provides more information than would be possible from using data only at 20 and 120 min. Single individual parameters measured or derived from in vitro digestion showed rather inconsistent relationships to the dose and types of added fibres or CPF. However, in vitro glucose release from starch could be modelled effectively with a modification of the Chapman–Richards model (see Online Resource) described by van Van Kempen [30]. With this model, the rate of starch digestibility (k) can be estimated, and together with %RDS, AUC 120 and the carbohydrate level in a regression model there was a very high correlation with in vivo plasma glucose responses.

There are some limitations of the study that may affect direct extrapolation to other situations. The study population has a very high proportion of women. However, we did not have any a priori selection criteria or hypotheses relating to gender, and previous research suggests gender is not a significant contributor to between-food variation in glycemic responses [61]. For some treatments, the study was underpowered due to unequal numbers of dropouts across treatment sequences. In addition, specific sources of GG and KM have been used and other types of GG or KM could possibly influence the glycaemic response differently. One of the strengths of the study is that it is well controlled as the incomplete block design allows for all the comparisons to be executed within one study period and population.

Taken together, these data demonstrate that flatbread flour mixes supplemented with specific combinations of GG and CPF or higher concentrations of GG of KM alone can produce marked, significant reductions in PPGs (i.e. ≥30 % reduction in +iAUC) in healthy adults. The results also show that a model using a range of in vitro measured parameters may be a useful predictive tool for in vivo PPG responses to these ingredients in this product format.

## Electronic supplementary material

Below is the link to the electronic supplementary material.

Supplementary material 1 (PDF 377 kb)

## Abbreviations

CPF: Chickpea flour
*C*_max_: Maximum observed glucose response
GG: Guar gum
GI: Glycaemic index
GL: Glycaemic load
HFF: High-fibre flour
KM: Konjac mannan
PPG: Post-prandial plasma glucose
+iAUC: Positive incremental area under the curve
RDS: Rapidly digestible starch
RS: Resistant starch
SDS: Slowly digestible starch
tAUC: Total area under the curve
*T*_max_: Time at which the *C*_max_ is reached
T2DM: Type 2 diabetes mellitus
