# Supplementary material for: Efficacy of different fibres and flour mixes in South-Asian flatbreads for reducing post-prandial glucose responses in healthy adults
Source: Eur J Nutr. 2016 Jun 21;56(6):2049–60. doi: 10.1007/s00394-016-1242-9 (PMC5579182; doi:10.1007/s00394-016-1242-9)

Online Resource:

Article title: Efficacy of different fibres and flour mixes in South-Asian flatbreads for reducing post-prandial glucose responses in healthy adults.

Journal Name: European Journal of Nutrition

Author names: Hanny M. Boers, Katrina MacAulay, Peter Murray, Jack Seijen ten Hoorn, Anne-Roos Hoogenraad, Harry P.F. Peters, Maria A.M. Vente-Spreeuwenberg & David J. Mela

Corresponding author: Hanny M. Boers, Unilever R&D Vlaardingen, PO Box 114, 3130 AC Vlaardingen, The Netherlands. e-mail: hanny.boers@unilever.com

Supplemental Table 1. Selection criteria

Age at start of the study ≥ 20 and ≤ 50 years
Body mass index (BMI) ≥ 20 and ≤ 25.0 kg/m^2
Apparently healthy: no medical conditions which might affect study measurements (judged by study physician or measured by questionnaire).
Having a general practitioner.
No use of medication which interferes with study measurements (as judged by the study physician).
Agreeing to be informed about medically relevant personal test-results by a physician
Informed consent signed
No reported participation in another nutritional or biomedical trial 3 months before the pre-study examination or during the study.
Willing to comply to study protocol during study.
No reported participation in night shift work two weeks prior to pre-study investigation or during the study. Night work is defined as working between midnight and 6.00 AM
Reported intense sporting activities ≤ 10h/w
Consumption of =< 14 alcoholic drinks in a typical week (females) or =< 21 alcoholic drinks in a typical week (males).
Currently not smoking and being a non-smoker for at least six months and no reported use of any nicotine containing products in the six months preceding the study and during the study itself.
Not being pregnant during the study period or in the six months prior to pre-study investigation and no intention to become pregnant.
No reported lactating 6 weeks before pre-study investigation and during the study.
Reported dietary habits: no medically prescribed diet, no slimming diet, used to eat 3 meals a day, no vegetarian.
Not reported weight loss/gain (>10%) in the last six month before the study
Not being an employee of Unilever or Leatherhead.
No dislike, allergy or intolerance to test products.
A fasting blood glucose value between 3.5 - 5.6 mmol/litre at screening (This will be measured by finger prick).
No blood donation 1 month (males) or 2 months (females) prior to pre-study examination or during the study.

Supplemental table 2: Subject baseline characteristics by gender (mean ± SD)

**Gender**	**N**	**Variable**	**Mean**	**SD**
**Female**	35	Age (yr)	36.60	8.92
		Height (m)	1.66	0.07
		Weight (kg)	63.07	6.61
		BMI (kg/m^2)	22.88	1.50
		Fasting glucose (mmol/l)	5.11	0.38
**Male**	3	Age (yr)	42.00	11.36
		Height (m)	1.83	0.14
		Weight (kg)	76.33	20.32
		BMI (kg/m^2)	22.35	2.47
		Fasting glucose (mmol/l)	5.35	0.14

In vitro starch digestion method

In vitro starch digestibility was assessed by adaptations of the Englyst method [26, 27], with which good correlations have been demonstrated between clinical postprandial glucose (PPG) responses and the in vitro starch digestibility measures of rapidly digestible, slowly digestible and resistant starch (RDS, SDS and RS) for a wide range of products [28]. The method was modified with the methods described by Sopade [29] and Van Kempen [30] to provide a glucose release profile and calculate the rate of digestion (k) using the Chapman-Richards model and the Area under the glucose curve over 120 min (AUC120) using the trapezoidal model.

A detailed description of the experimental conditions of all models is given in Supplemental Table 2. Our model ('URDV') was tested with the same types of food as used by Englyst et al [28], and Supplemental Figure 1 shows comparable results for RDS and SDS. Our model was also tested with reference materials used by Van Kempen et al [30] and Supplemental Table 2 lists the obtained parameters.

The additional inclusion of the simulated oral digestion stage by 30 seconds mixing with α-amylase will increase RDS and k. The chewing and wetting with saliva of the flatbreads is seen as an essential part of starch digestion. Furthermore the pH of the intestinal phase has been increased from 5.2 to 6.5, to reflect realistic human physiology.

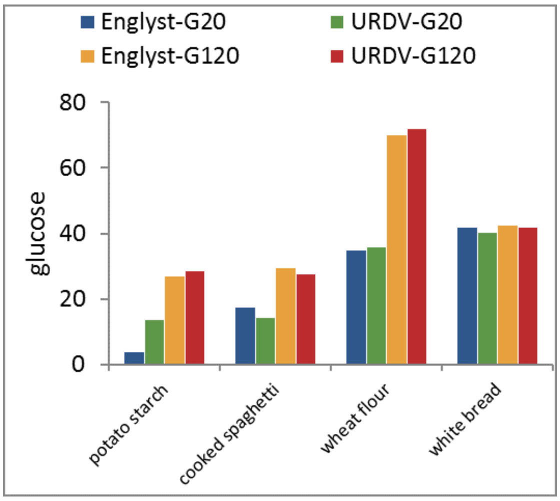

Supplemental Figure 1 Reference materials according to Englyst (1999), with comparison of % available glucose released at 20 (G20) and 120 (G120) min for that and the modified (URDV) model.

Supplemental Table 3 Experimental conditions of *in vitro* starch digestibility methods

	Englyst [28]	Sopade [29]	Van Kempen [30]	URDV*
Number of repeats			Triplicate	Duplicate
Sample volume [g]	<0.6g carb	0.5	1	0.5
Sample treatment	Mincer with 0.9cm holes		Retsch grinder ZM1, 1mm screen	Sliced chapatti, 3x7mm
Tube volume [ml]	50		50	50
Mouth simulation	-	15-20s treatment with 1ml α-amylase	-	Vortex mixed, 1ml α-amylase, 30s
α-amylase activity		Sigma A-3176 Type VI-B; 250 U per ml of carbonate buffer		6.5mg α-amylase Sigma 10080, 300U
Stomach simulation	Vortex mixed, 30min 37°C in water bath 160 strokes/min	30min, 37°C in a water bath (SWB20) at 85 rpm	30min, 39°C, horizontal agitation with glass beads	30min, 37°C, horizontal agitation with 5 glass beads, 10mm diameter
Enzymes	50mg pepsin Sigma P7000, 13kU	1ml Sigma P6887	50mg pepsin Sigma P7000, 13kU	25mg pepsin Sigma P77160, 16kU

Gastric solution	15ml: 200mg arabinose, 50mg guar gum in 0.05M HCl	5ml: 0.02M HCl	10ml: 50mg guar gum in 0.05M HCl	5ml: 25mg guar gum in 0.05M HCl
Neutralisation	5ml 0.5M Na-acetate, pH 5.2, add 5 glass balls (15mm) shake at 37°C for few min.	5ml 0.02M NaOH 25ml 0.2M Na-acetate, pH 6.0	10ml 0.25M Na-acetate	1ml 0.25M NaOH 25ml 0.2M acetate (incl. 0.15M NaCl, 5mM KCl, 5mM $CaCl_2$), pH 6.0
Intestine simulation	Shake in water bath (160 strokes/min)	240min, 37°C	480min, 39°C, horizontal agitation	240min, 37°C, horizontal agitation
Enzymes	5ml solution: 675mg Pancreatin Sigma P7545, 8xUSP 0.2ml AMG 400L Novo Nordisk, 80U 0.3ml Invertase Merck 390203D,	5ml acetate solution: 10mg pancreatin Sigma P1750 amyloglucosidase Sigma A7420 from Aspergillus niger; 140U	5ml aqueous solution 700mg pancreatin Sigma P7545, 8xUSP 0.05ml AMG Englyst 61-002, 10U 3mg invertase Sigma P57629, 500U	5ml acetate solution: 56mg pancreatin Sigma P1625, 3xUSP 0.33ml AMG Sigma A7095, 100U 0.33ml invertase BDH39020, 1000EU
Total volume [ml]	25	41	25	37
Sampling volume [ml]	0.2	0.006	0.5	0.2
Enzyme inactivation	4ml 100% ethanol	-	100% ethanol	4.8ml 80% ethanol
Sampling [min]	20 (RDS), 120 (SDS)	0, 10, 20, 30, 45, 60, 90, 120, 150, 180, 210, 240	0, 15, 30, 60, 120, 240, 360, 480	20, 60, 120, 180, 240
Glucose analysis	HPLC	Accu-Check Performa glucometer	Glucose oxidase kit, Megazyme	Glucose oxidase kit, Sigma

*URDV = Unilever Research and Development Vlaardingen, Vlaardingen, The Netherlands

Supplemental table 4 Starch digestion parameters for reference materials according to Van Kempen

	**Van Kempen**			**URDV***		
	Waxy rice starch	Rice starch	Nastar pea starch	Waxy rice starch	Rice starch	Nastar pea starch
Plateau [% starch]	75.9	74.8	73.9	73.4	67.5	53.1
Rate of digestion (k) [% starch/min]	1.92	1.02	0.38	5.8	0.86	0.47
RDS	28.8	19.7	7.1	73.6	27.1	2.9
SDS	68.1	46.8	31.7	26.4	29.2	14.9
RS	3.1	33.5	61.2	0	43.7	82.3

*URDV = Unilever Research and Development Vlaardingen, Vlaardingen, The Netherlands

The amount of glucose, released during intestinal digestion, is expressed as fraction of the total carbohydrates in the chapatti. The carbohydrates content is estimated using the calculation:

$$c = \frac{W_f \times c_f \times W_{db}}{W_d \times W_c}$$

In which:
- W_f = weight of flour mixture
- c_f = carbohydrates content of flour mixture
- W_{db} = weight of dough ball
- W_d = weight of dough
- W_c = weight of chapatti

Since most of the digestion of chapatti takes place within the first 60 minutes, the initial rate of digestion would provide an additional parameter to discriminate between treatments. Therefore, non-linear regression was applied using the Chapman-Richards model as suggested by Van Kempen, as:

$$Glu\cos e(t) = A + B \times \left[1 - \exp\left(-K/(A+B) \times time\right)\right]^{(C/(A+B)+1)}$$

In which:
- A = free glucose present in the sample before enzyme addition
- B = glucose released by exhaustive digestion
- K = rate of glucose release corrected for plateau effects
- C = sigmoidal/shape modifier corrected for plateau effects.

The fitted glucose curve for time (t) is 0 to 120 minutes is used to calculate the AUC120 using the trapezoidal model with the equation:

$$\int_{t0}^{t120} Glu\cos e(t) = \sum \left(\frac{glu_2 - glu_1}{2}\right)\left(t_2 - t_1\right)$$

The in vitro starch digestion parameters are listed in Supplemental Table 3.

Supplemental Table 5 Measured starch digestion parameters of chapattis

Chapatti composition	**Carb** [%]	**k** [g/min]	**AUC**	**RDS** [%]	**SDS** [%]	**RS** [%]
Hi-Fibre Flour (HFF control) Atta	61.0	3.45	9150	48.2	38.7	13.1
81% HFF + 15% CPF + 4% GG	54.7	3.83	8887	53.7	37.7	8.6
83% HFF + 15% CPF + 2% GG	55.9	4.17	8461	53.0	32.1	14.9
85% HFF + 15% CPF	57.1	4.47	8563	54.8	30.9	14.3
86% HFF + 10% CPF+ 4% GG	56.0	3.57	8441	50.5	36.4	13.0
88% HFF + 10% CPF + 2% GG	57.2	3.79	8399	51.6	33.0	15.3
94% HFF + 6% GG	57.3	3.14	8433	48.1	46.2	5.7
96% HFF + 4% GG	58.6	3.83	8453	52.0	34.2	13.9
98% HFF + 2% GG*	59.8	4.44	8461	54.4	30.2	15.3
96% HFF + 4% KM	58.6	4.06	8015	51.3	29.1	19.6
98% HFF + 2% KM	59.8	3.14	8333	47.6	41.0	11.3
Market Standard Atta	64.2	4.28	9104	63.7	30.8	5.6

*GG = guar gum; KM = konjac mannan; CPF = chickpea flour
Carb= carbohydrate content of flour mix
K = rate of digestion from Chapman-Richards model
AUC = area under the curve over 120 minutes
RDS = rapidly digestible starch
SDS = slowly digestible starch
RS = resistant starch

In vitro prediction of in vivo data

The aim of the statistical analysis was to identify the best model to predict +iAUC over 120 minutes in the in vivo study from the in vitro data. The PPG response in the clinical trial are shown in the Table 3 of the main article text. All parameters that describe the in vitro digestibility (listed in Supplemental Table 4 above) plus 'slope to Cmax' were considered as input for correlation with the *in vivo* data. Initial correlations and scatter plots for each of these variables with +iAUC showed that a linear model was unlikely to provide good prediction, so a quadratic model was used.

The best model was determined to be one including carbohydrate level, k**,** %RDS and AUC over 120 minutes. This model had R2 = 0.97 and adjusted R2 = 0.89.

The full resulting model was:

+iAUC = 31417 + 334.6*CHO - 9729*k - 4.739*AUC_*in vitro* - 32.03*RDS +18.38*CHO*k *1.183*k*AUC_*in vitro* – 3.542*CHO^2 – 132.2*k2

where k = rate of digestion from Chapman-Richards model; AUC *in vitro* = AUC for 120 minutes *in vitro*; CHO = carbohydrates content of flour mix**;** RDS = rapidly digestible starch

For this particular format and the selected fibre mixes a modified model using only the Englyst parameters SDS, RDS and RS (in fact SDS and RS only, because RDS and SDS cannot be fitted together) was:

+iAUC = -79.60*SDS -3.93*RS +0.95*SDS*RS +0.785*SDS^2 -1.37*RS^2
This gave R^2 = 0.61 and an adjusted R^2 = 0.28, which was much lower than the selected model described above

Supplemental Figure 2: Satiety scores, tAUC/120 min, change from control (all mean +/- 95% CI)

Supplemental Figure 2a: How hungry are you?, tAUC/120 min, change from control (mean +/- 95% CI)

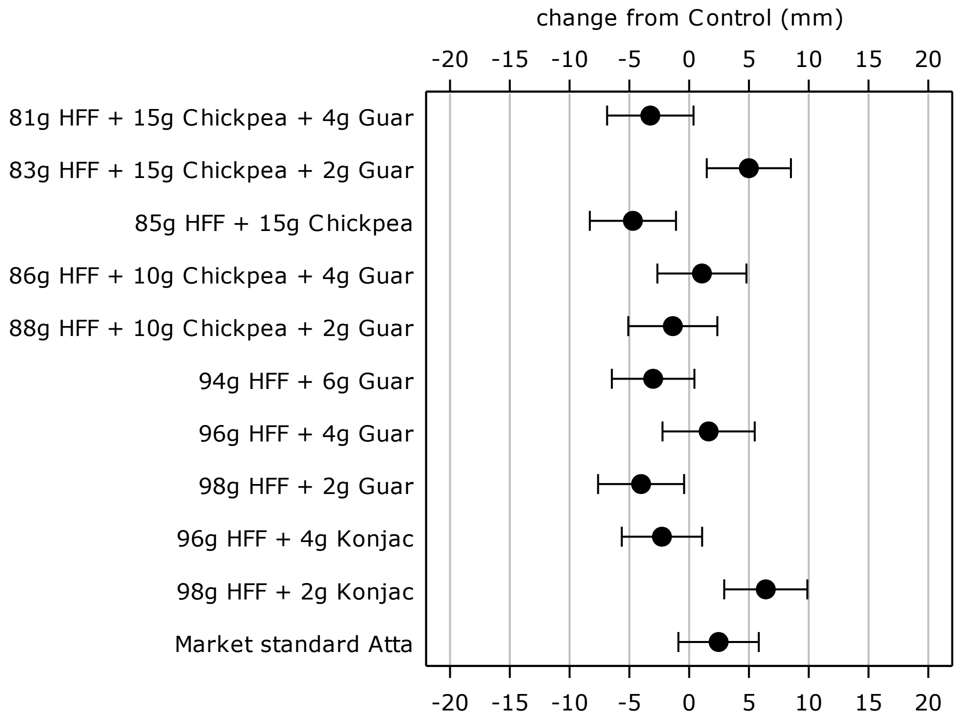

Supplemental Figure 2b: How full are you?, tAUC/120 min, change from control (mean +/- 95% CI)

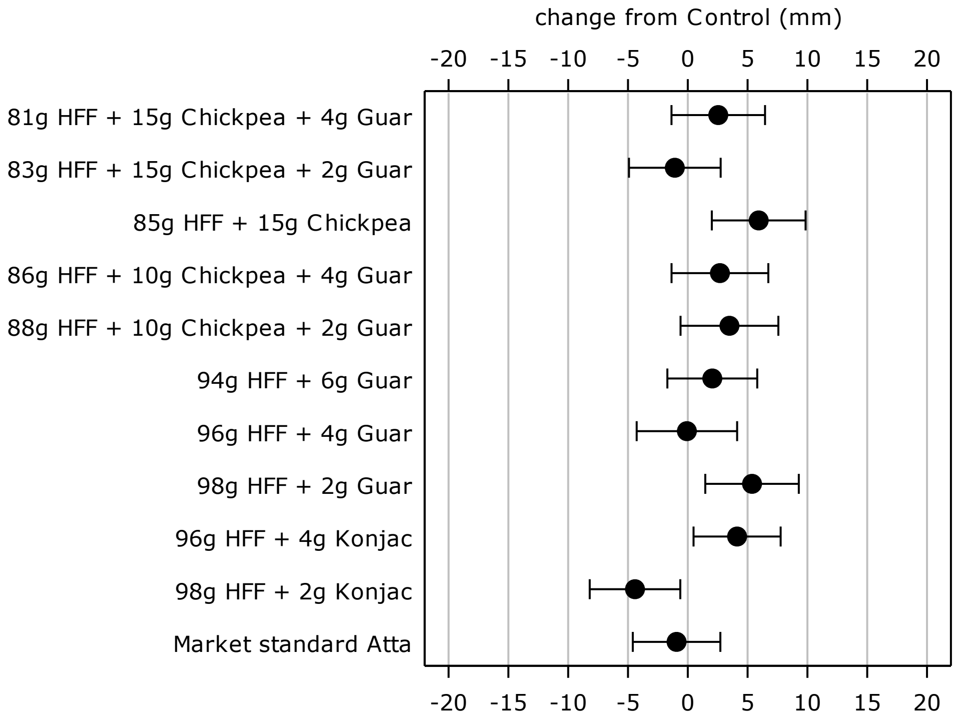

Supplemental Figure 2c: How strong is your desire to eat a meal?, tAUC/120 min., change from control (mean +/- 95% CI)

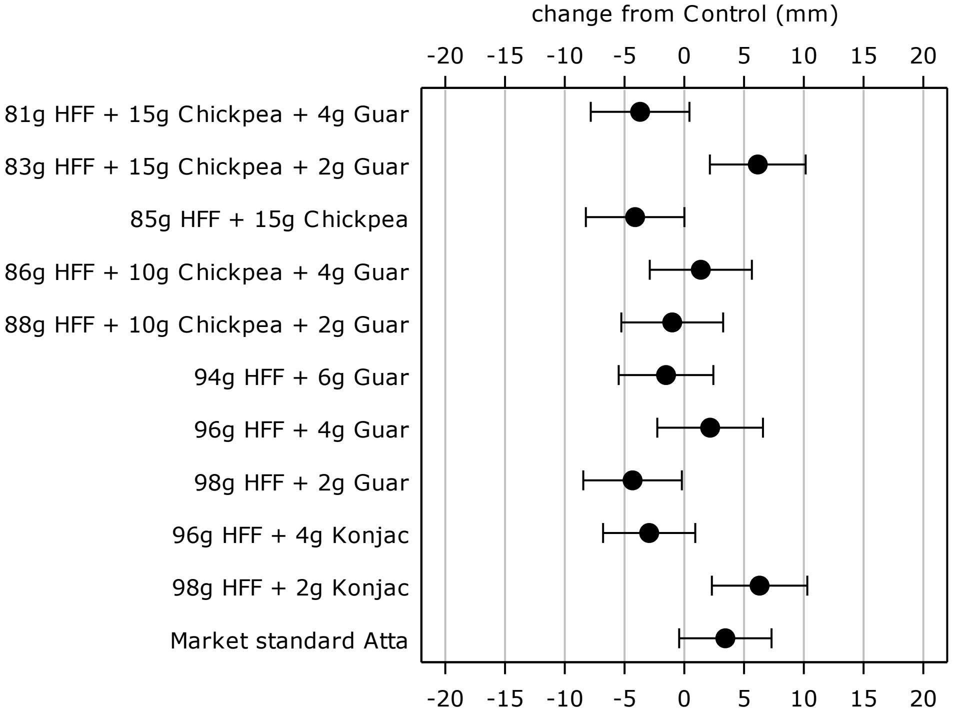

Supplement: Supplementary file 1 — Supplementary material 1 (PDF 377 kb) [file 394_2016_1242_MOESM1_ESM.pdf]
